# Supplementary material for: Integrative Genomics and Metabolomics Analyses Provide New Insights into the Molecular Basis of Plant Growth Promotion by Pantoea agglomerans
Source: Microorganisms. 2025 Sep 12;13(9):2138. doi: 10.3390/microorganisms13092138 (PMC12472927; doi:10.3390/microorganisms13092138)
Supplement: Supplementary file 1 [file microorganisms-13-02138-s001.zip › microorganisms-3787330-supplementary/Supplementary microorganisms-3787330/Figure S1.pdf]

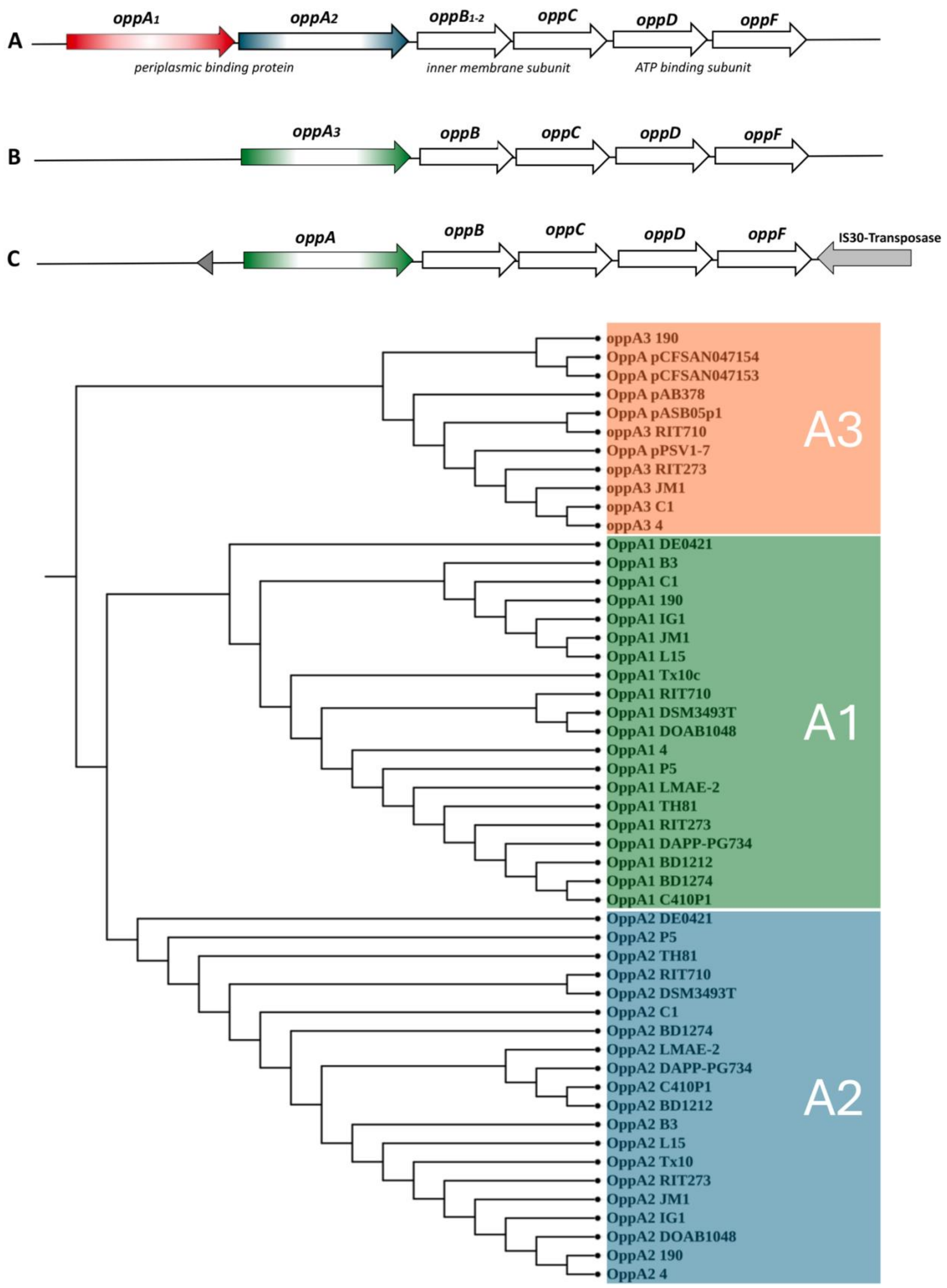

Figure S1. Structure of the primary (A) and the accessory (B) oligopeptide transporter *opp* operons from *P. agglomerans*. Line C illustrates the structure of the highly similar *opp* operons found in *P. agglomerans* plasmids, which contain a site-specific IS30 integrase. The dendrogram shows the consensus phylogenetic tree of the *oppA* proteins from *P. agglomerans*. The alignment of protein sequences was carried out using ClustalW. The tree topology reveals the presence of three clades, designated as A1-A3.
