# Supplementary material for: Integrative Genomics and Metabolomics Analyses Provide New Insights into the Molecular Basis of Plant Growth Promotion by Pantoea agglomerans
Source: Microorganisms. 2025 Sep 12;13(9):2138. doi: 10.3390/microorganisms13092138 (PMC12472927; doi:10.3390/microorganisms13092138)
Supplement: Supplementary file 1 [file microorganisms-13-02138-s001.zip › microorganisms-3787330-supplementary/Supplementary microorganisms-3787330/Figure S2.pdf]

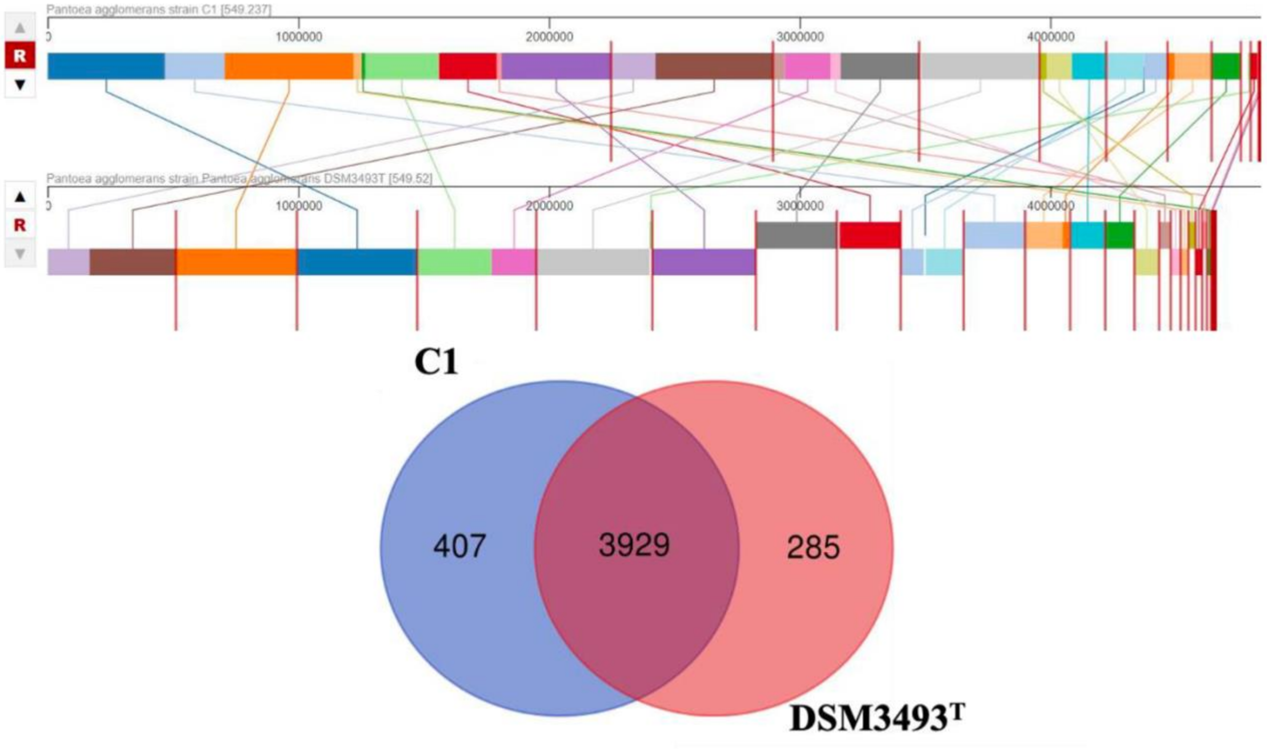

Figure S2. Comparison of *P. agglomerans* strain C1 and DSM3497^{T} genomes. The upper part shows the alignment of the two genomes obtained using the progressive MAUVE method. Each colored rectangle represents a locally collinear block or homologous region shared between the genomes, and colored lines indicate aligned segments between them. The Venn diagram below illustrates shared (burgundy) and unique proteins of the two *P. agglomerans* strains, blue for strain C1 and peach for strain DSM3493^{T}.
