# Supplementary figures and images for: Integrative Genomics and Metabolomics Analyses Provide New Insights into the Molecular Basis of Plant Growth Promotion by Pantoea agglomerans
Source: Microorganisms. 2025 Sep 12;13(9):2138. doi: 10.3390/microorganisms13092138 (PMC12472927; doi:10.3390/microorganisms13092138)

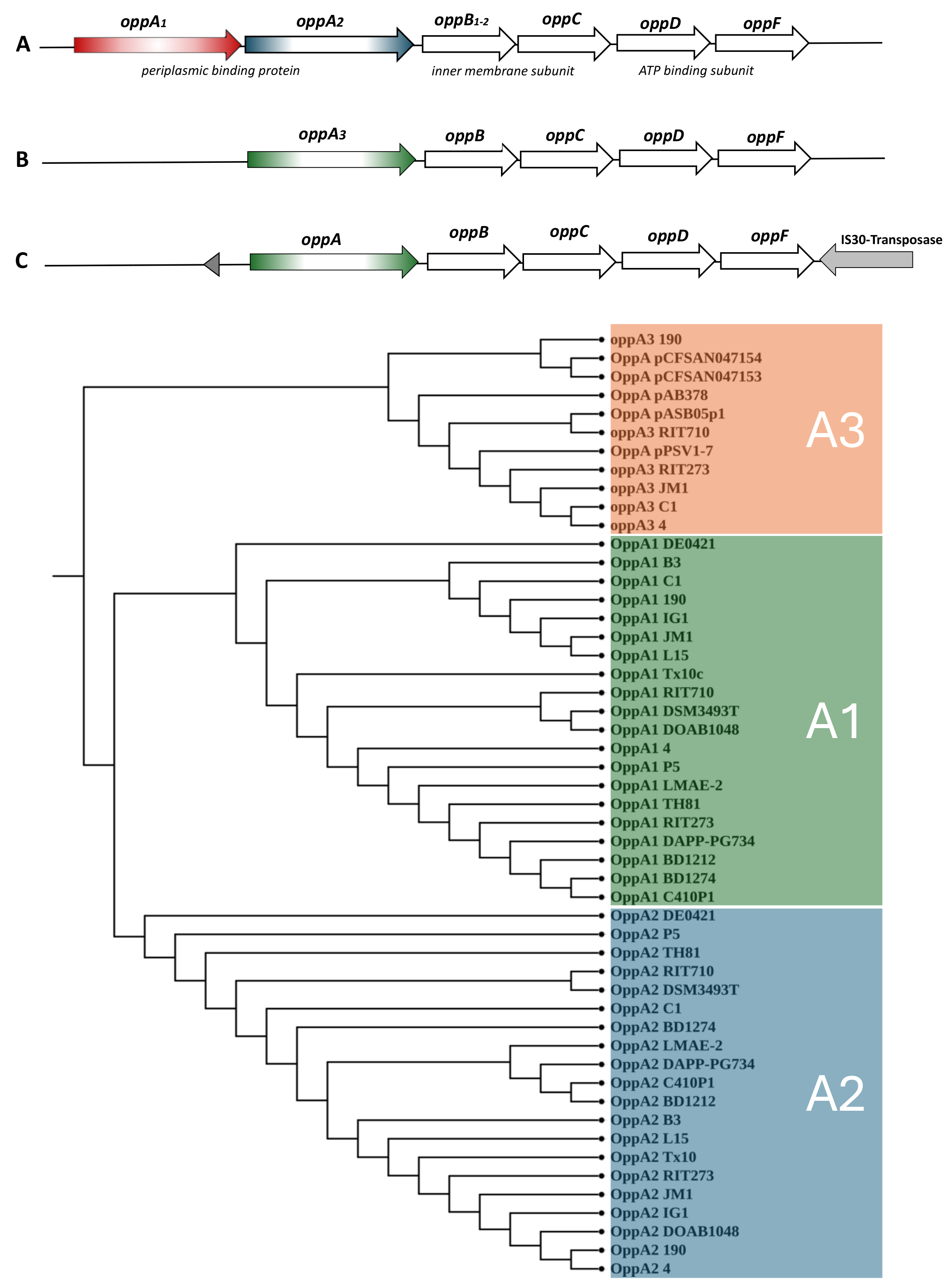

Supplement: Supplementary file 1 [file microorganisms-13-02138-s001.zip › microorganisms-3787330-supplementary/Supplementary microorganisms-3787330/Figure S1.tif]

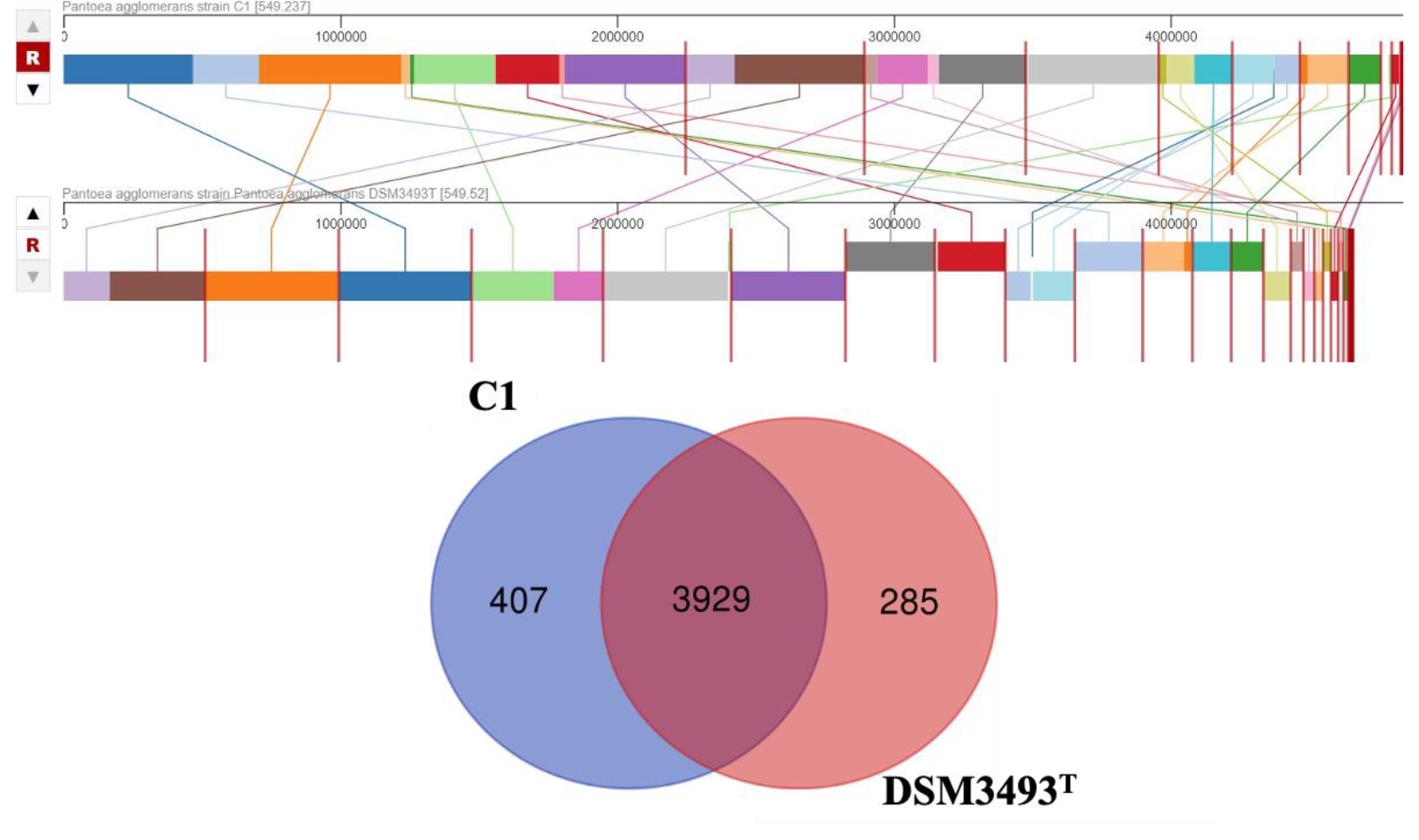

Supplement: Supplementary file 1 [file microorganisms-13-02138-s001.zip › microorganisms-3787330-supplementary/Supplementary microorganisms-3787330/Figure S2.tif]
